# Recent Advances in Photodynamic Therapy against Fungal Keratitis

**DOI:** 10.3390/pharmaceutics13122011

**Published:** 2021-11-26

**Authors:** Jia-Horung Hung, Chaw-Ning Lee, Huai-Wen Hsu, I-Son Ng, Chi-Jung Wu, Chun-Keung Yu, Nan-Yao Lee, Yun Chang, Tak-Wah Wong

**Affiliations:** 1 Institute of Clinical Medicine, College of Medicine, National Cheng Kung University, Tainan 701, Taiwan; hungjh@mail.ncku.edu.tw; 2 Department of Ophthalmology, National Cheng Kung University Hospital, College of Medicine, National Cheng Kung University, Tainan 704, Taiwan; 3 Department of Dermatology, National Cheng Kung University Hospital, College of Medicine, National Cheng Kung University, Tainan 704, Taiwan; Joyce060324@gmail.com; 4 Institute of Clinical Pharmacy and Pharmaceutical Sciences, College of Medicine, National Cheng Kung University, Tainan 701, Taiwan; 5 School of Medicine, National Cheng Kung University, Tainan 701, Taiwan; haley3006@gmail.com (H.-W.H.); c.phoebe.y@gmail.com (Y.C.); 6 Department of Chemical Engineering, National Cheng Kung University, Tainan 701, Taiwan; yswu@mail.ncku.edu.tw; 7 National Institute of Infectious Diseases and Vaccinology, National Health Research Institutes, Tainan 704, Taiwan; wucj@nhri.edu.tw; 8 Division of Infectious Diseases, Department of Internal Medicine, National Cheng Kung University Hospital, College of Medicine, National Cheng Kung University, Tainan 704, Taiwan; nanyao@mail.ncku.edu.tw; 9 Institute of Basic Medical Sciences, College of Medicine, National Cheng Kung University, Tainan 701, Taiwan; dckyu@ncku.edu.tw; 10 Department of Microbiology and Immunology, College of Medicine, National Cheng Kung University, Tainan 701, Taiwan; 11 Department of Biochemistry and Molecular Biology, College of Medicine, National Cheng Kung University, Tainan 701, Taiwan; 12 Center of Applied Nanomedicine, National Cheng Kung University, Tainan 701, Taiwan

**Keywords:** candida, collagen cross-linking, drug delivery, fungal infection, flavin mononucleotide, keratitis, rose bengal, photodynamic therapy, drug-resistance

## Abstract

Fungal keratitis is a serious clinical infection on the cornea caused by fungi and is one of the leading causes of blindness in Asian countries. The treatment options are currently limited to a few antifungal agents. With the increasing incidence of drug-resistant infections, many patients fail to respond to antibiotics. Riboflavin-mediated corneal crosslinking (similar to photodynamic therapy (PDT)) for corneal ectasia was approved in the US in the early 2000s. Current evidence suggests that PDT could have the potential to inhibit fungal biofilm formation and overcome drug resistance by using riboflavin and rose bengal as photosensitizers. However, only a few clinical trials have been initiated in anti-fungal keratitis PDT treatment. Moreover, the removal of the corneal epithelium and repeated application of riboflavin and rose bengal are required to improve drug penetration before and during PDT. Thus, an improvement in trans-corneal drug delivery is mandatory for a successful and efficient treatment. In this article, we review the studies published to date using PDT against fungal keratitis and aim to enhance the understanding and awareness of this research area. The potential of modifying photosensitizers using nanotechnology to improve the efficacy of PDT on fungal keratitis is also briefly reviewed.

## 1. Introduction

According to the World Health Organization (WHO), around 6 million people globally are affected by cornea-related blindness [1]. Corneal opacity is estimated to be responsible for 1.5–2.0 million new cases of monocular blindness each year [1], with etiologies including infection, trauma, inflammation, degeneration, and nutritional deficiency [1]. Among the etiologies, infectious keratitis (IK) stands at the top with an estimated incidence of 2.5–799 per 100,000 population-year [2]. IK can be caused by pathogens, such as bacteria, fungi, virus, parasites, and polymicrobial infections, which may vary depending on different geographic locations and seasons [3].

Bacterial infections make up 79–100% of IK, depending on the country and study period [1]. Fungal keratitis, on the other hand, is more prevalent in Asian countries [2,3]. It is a serious corneal fungal infection, commonly caused by *Candida*, *Fusarium,* and *Aspergillus,* that often results in blindness and eye loss, especially in developing countries [4]. The global minimal annual incidence is estimated at 1.05 million cases, with the highest rates in Asia and Africa. Even with the advancement of biotechnology, there are few antifungal agents available, including natamycin, amphotericin B, fluconazole, and voriconazole [1]. The situation is complicated by the rapid emergence of drug-resistant fungal keratitis globally [5,6], to the extent that some patients require a full thickness corneal transplantation (penetrating keratoplasty) [7] as treatment. In their 1991 study, Kirkness et al. suggested early intervention with corneal transplantation regarding the management of advanced microbial keratitis [8]. The overall success rate is around 80–90% [7,9], however graft failure and the recurrence of infection could occur in an active infected eye after operation [10]. Furthermore, the acquired and innate antifungal drug resistance has drastically increased over the past three decades [2]. Moreover, the clinical response to fungal infection does not always correlate with in vitro drug sensitivity testing [11,12]. Hence, new and novel therapies are crucially required to treat and prevent drug-resistant fungal infections.

Photodynamic therapy (PDT) comprises the activation of a specific photosensitizer (PS) with an absorption peak light wavelength of the PS in the presence of oxygen molecules in the tissue and has been widely used to kill cancer cells for three decades [13]. The application of PDT against microorganisms can be dated back to the 1900s when Rabb showed photodynamic effects after exposing *Paramecium caudatum* to acridine or eosin dyes and illuminated them with sunlight [14]. Even though antimicrobial PDT (aPDT) has shown great potential in treating drug-resistant infectious diseases in vitro and in animal studies, only a few clinical trials are currently ongoing [14,15]. Yet, aPDT has several advantages: (1) It is a local treatment with extremely rare systemic side effects; (2) The antimicrobial effects are medicated by the generation of singlet oxygen and reactive oxygen species (ROS) during irradiation, which damage multiple organelles in a cell, thus PDT resistance has not yet been reported; (3) It functions well both in targeting against planktonic and in biofilm microorganisms [14,15]; (4) Bacteria survive after PDT reduced resistance to antibiotics [16], and some PSs bind more rapidly and selectively to microbials than human cells [14]. So, the killing of the microbials is highly selective in aPDT.

In the field of ophthalmology, PDT was introduced to treat choroidal neovascularization in the 1990s [17]. Before then, the role of PDT in eliminating ocular infection had been rarely studied. Riboflavin-mediated corneal crosslinking (CXL), which is a form of PDT, utilizes riboflavin eye drops as a PS and activates with ultraviolet-A (UVA) to increase the stiffness of the cornea [18]. After its introduction in 2003 by Theo Seiler [18], the application of CXL was extended to IK [19]. Recently, aPDT that utilizes rose bengal as a PS and activation with green light has shown a 72% success rate in IK patients [20,21].

In this review, we focus on the advancement of aPDT against fungal keratitis, to spotlight a less studied area and enhance the awareness of this area of translational studies.

## 2. Fungal Keratitis

Fungal keratitis was first described by Leber in 1879. It is a serious corneal infection with poor visual prognosis [1,22,23,24], causing a significant socioeconomic burden, especially in developing countries because it commonly affects young male outdoor agricultural workers [25]. The incidence of fungal keratitis has increased over the past three decades due to the frequent use of topical corticosteroids and antibiotics in IK treatment. The estimated minimum annual incidence is around 1 million worldwide with the highest rates in Asia and Africa, and the loss of around 84,143–115,697 eyes [4]. The proportions of fungal keratitis in IK vary from less than 10% in temperate regions to more than 45% in tropical and subtropical regions [4]. The most common pathogens causing fungal keratitis are filamentous fungi (*Fusarium*, *Aspergillus*) and yeasts (*Candida albicans* and other *Candida* species) [1]. Fungi enter corneal stroma through the epithelial defect or extend from the posterior segment through the descent membrane (fungal endophthalmitis). Another entry pathway is through corneoscleral trabeculae into the corneal channel network, since trauma to the corneal epithelium by a contaminated sharp object is very common in farmers in developing countries. In addition to trauma, risk factors for fungal keratitis include pre-existing ocular disorders, systemic disorders, wearing of contact lenses, topical steroid use, and recent ocular surgery [9].

The treatment of fungal keratitis remains challenging because of the difficulty in early diagnosis, limited choices in anti-fungal agents, the emergence of antifungal drug tolerance and resistance [6], and the formation of biofilm, which will be further elaborated in the following section. The mainstay medical treatment is topical anti-fungal agents, e.g., polyenes (amphotericin B, natamycin), triazoles (fluconazole, voriconazole, posaconazole), echinocandins (caspofungin, micafungin), and pyrimidine analogue (flucytosine) with or without systemic antifungal agents [22,24]. Fusarium keratitis is difficult to treat because the *Fusarium* spp. are intrinsically resistant to most antifungals, including echinocandins [26,27]. Since the approval of natamycin in the 1960s by the US Food and Drug Administration, no new topical antifungal eye drops have been approved and natamycin is currently considered the most effective medication against *Fusarium* [24].

Among the available antifungal agents, voriconazole has demonstrated the best ocular penetration and broadest coverage of fungal species in vitro [23]. To overcome the disadvantage of poor corneal penetration of antifungal agents, intrastromal or intracameral drug injections have also been proposed [28]. Even with the advancement of new drugs and a new methodology, 40–60% of fungal keratitis cases are refractory to medical therapy and require surgical intervention [9,22], including multiple keratectomies or penetrating keratoplasty. For patients receiving therapeutic keratoplasty performed in an active infection stage, the five-year survival rate was only 51% compared to 90% in cases with inactive infection [8]. Moreover, long-term use of immunosuppressants can lead to repetitive *Candida* keratitis, which may require multiple corneal transplantations [29]. Overall, fungal keratitis is associated with poorer visual outcomes and remains a great challenge to ophthalmologists [22,24,26].

## 3. Drug Resistance and Biofilm in Fungal Keratitis

The emergence of drug resistance in fungal infection poses a significant threat to public health globally [5,6,30]. Cases of multidrug resistant *Fusarium* keratitis [12,30,31,32] or azole-resistant *Candida* keratitis [33] are rare but can be very challenging once they occur. In cases with multidrug-resistant fungal keratitis, the visual outcome is generally devastating despite intense conventional treatments, even requiring some patients to undergo enucleation to control the infection.

**Figure 1:**
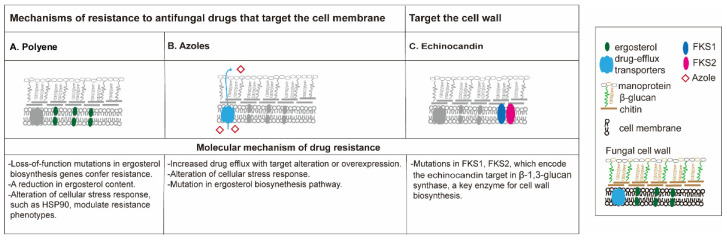
Mechanisms of antifungal agent resistance. Polyenes (**A**) and azoles (**B**) are membrane targeting antifungal drugs while echinocandins are cell wall-active agents. (**A**) Polyene resistance is often attributed to loss-of-function mutations in ergosterol biosynthetic genes which lead to depletion of ergosterol, the fungi-specific cell membrane sterol. Resistance mechanisms for *Candida albicans*, *Cryptococcus neoformans*, and *Aspergillus fumigatus* are outlined in dashes. (**B**) Azole resistance can result from the upregulation of two classes of efflux pumps that remove the drug from the cell; through the mutation or overexpression of *ERG11*, which minimizes the impact of the drug on the target; or alterations in ergosterol biosynthesis, such as the loss-of-function mutation of *ERG3*, which blocks the accumulation of a toxic sterol intermediate that is produced when *ERG11* is inhibited. (**C**) Resistance to echinocandins can result from mutations in *FKS1* that minimize the impact of the drug on the target.

The mechanism of antifungal drug resistance (Figure 1) is different among different classes of drugs [34,35]. The polyenes are the oldest class of antifungal drugs and include amphotericin B and nystatin. Polyene drugs target ergosterol, a fungal-specific sterol synthesized in the plasma membrane. The sequestration of ergosterol leads to the increase of membrane permeability, eventually causing cell death. Resistance to polyenes is rarely reported, and mainly related to decreased membrane ergosterol, an alteration of cellular stress response (mutations in *ERG3* gene) as reported in *Candida* [34,36]. In some cases, treatment with an azole antifungal, which in turn reduces ergosterol, can confer polyene resistance [36].

Azole antifungal agents are some of the most widely used antifungal agents, and offer activity against many fungal pathogens without the serious nephrotoxic effects observed with amphotericin B [37]. The azoles available in the clinic can be classified into two groups: the triazoles (fluconazole, itraconazole, voriconazole, posaconazole, and isavuconazole) and the imidazoles (ketoconazole). The azole antifungals are also membrane-targeted, primarily by inhibiting the cytochrome P450-dependent enzyme lanosterol 14-alpha-demethylase, a critical enzyme that converts lanosterol to ergosterol [38]. Triazole resistance is mainly caused by the increased activity of efflux pumps that remove the drug from the cell due to the overexpression or mutations of *ERG11* and *CYP51* genes, and/or the alteration of cellular stress response genes (loss-of-function of *ERG3* gene) [6,35].

Echinocandins constitute the first class of antifungals to target the fungal cell wall. This class of antifungals inhibits β-(1,3)-d-glucan synthase, a critical enzyme for the synthesis of polysaccharide β-(1,3)-d-glucan, a component of the cell wall of many fungi. Three semi-synthetic echinocandins, namely caspofungin, micafungin, and anidulafungin, have been developed for clinical use and are usually reserved for invasive fungal keratitis [39]. Clinical experience with this antifungal class suggests that it is among the best tolerated and safest classes of antifungals available [40]. The acquired resistance to echinocandins remains sporadic and varies by region but is possibly increasing, especially among invasive *C. glabrata* infections with *FKS1* and *FKS2* mutations [6].

A biofilm is defined as a structured microbial community attached to a surface and encased within a self-produced extracellular matrix [41,42], which blocks the entry of the antifungal agents [43]. Fungi isolated from keratitis are able to produce biofilm [44], impairing the susceptibility of antifungal agents, and protecting the fungi from UV light [44], thus enhancing fungal resistance [6]. The ability of fungi to form biofilms is correlated to their ability to form disease in humans [45], irrespective of the thickness of these biofilms [46]. Only a few antimycotics, such as miconazole (azoles), echinocandins, and liposomal formulations of amphotericin B (polyenes), have shown effectiveness against fungal biofilms [47,48]. Pérez-Laguna et al. reviewed the combination of aPDT and antimicrobial compounds to treat skin and mucosal infections in humans or animals [49]. They concluded that aPDT has additive or synergistic effects both in planktonic suspensions and biofilms, which may relate to an increase in membrane permeability by the aPDT in fluconazole-resistant *C. albicans* strains. Interestingly, combination therapies with natural products may enhance antifungal agents against biofilm. Lactoferricin B, a peptide of bovine lactoferrin exhibiting multiple biological functions, including antimicrobial, antiviral, antioxidant, and immunomodulatory activities, has been proposed to improve biofilm susceptibility to antifungals [50]. Other compounds, including monoterpenes, sesquiterpenes, extracts from microalgae, and Cyanobacteria, also showed enhancement of antifungal agents in fungal biofilm inhibition [51,52]. Their mechanisms are believed to relate to the induction of ROS by antifungal agents and targeting the fungal oxidative defense system [47]. Table 1 summarizes the treatment outcome of IK caused by multidrug-resistant fungi with traditional treatments.

## 4. The History of Antimicrobial Photodynamic Therapy

**Figure 2:**
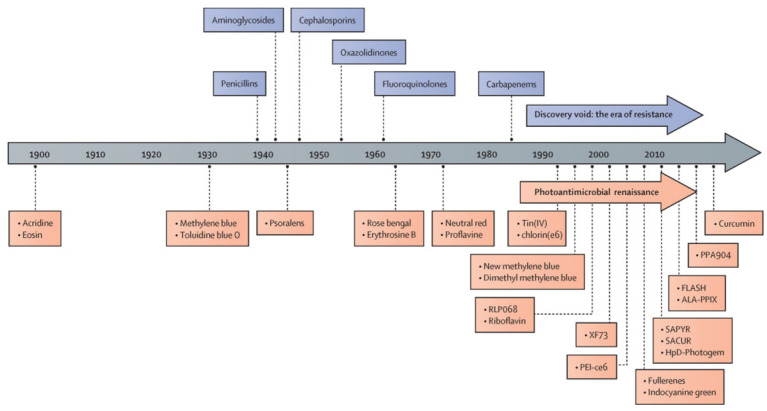
History of antimicrobial photodynamic therapy. RLP068: tetracationic Zn(II) phthalocyanine chloride; XF73: positively charged porphyrin; PEI-ce6: polyethyleneimine chlorin(e6) conjugate; SAPYR: perinapthenone derivative. SACUR: curcumin derivative; HpD-Photogem:haematoporphyrin derivative; FLASH: cationic riboflavin derivative; ALA-PPIX: 5-aminolevulinic acid-induced protoporphyrin IX; PPA90: tetrabutyl derivative of methylene blue. Reprinted from ref. [14] in text with permission from the Publisher.

Photodynamic therapy (PDT) has been used as a noninvasive treatment for the selective destruction of pathogenic organisms using a handful of non-toxic PSs since its earliest development (Figure 2). After the discovery of penicillin in 1928, the golden era of antibiotics began in the 1940s and lasted until late 1960s with the development of different classes of antibiotics, including aminoglycosides, tetracyclines, chloramphenicols, sulfones, macrolides, glycopeptides, polymyxins, oxazolidinones, ansamycins, quinolones, azoles, and ethambutol [15]. Similarly, the discovery of aPDT has been accelerated by the development of new classes of PSs since the 1900s, nowadays known as the era of drug resistance (Figure 2). The PSs investigated during the era of aPDT renaissance include tetracationic Zn(II) phthalocyanine PS (RLP-068) [57], methylene blue [58], Tin(IV) porphyrins [59], chlorine e6 [60], new formulations of methylene blue [61], riboflavin [62], exeporfinium chloride (XF73) [63], fullerenes [64], indocyanine green [65], 2-((4-pyridinyl)methyl)-1H-phenalen-1-one chloride (SAPYR) [66], curcumin derivative (SACUR-3) [67], hematoporphyrin derivative-Photogem [68], 5-aminolevulinic acid induced protoporphyrin IX (ALA-PpIX) [69], C₂₈H₄₂BrN₃S, phenothiazin-5-ium, 3,7-bis(dibutylamino)-, bromide (PPA904) [70], and curcumin [71].

## 5. Mechanism of the Photodynamic Action in Fungal Infection

**Figure 3:**
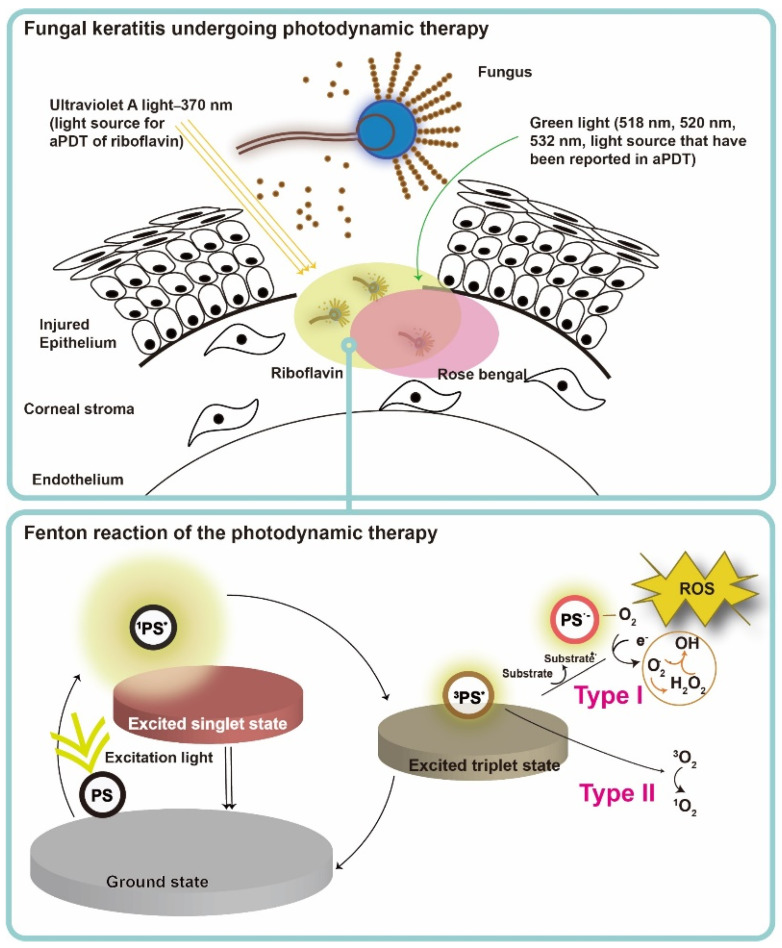
Schematic illustration of antimicrobial photodynamic therapy mechanism for fungal keratitis. The ground-state photosensitizer (PS) absorbs photons and is excited to the first short-lived excited singlet state and either returns to the ground state or undergoes intersystem crossing to a long-lived triplet state. The triplet state PS exerts downstream function via a type I or type II photosensitization process. For type I reaction, charge is transferred from the excited PS to oxygen (O_2_), and therefore leading to the formation of hydrogen peroxide (H_2_O_2_), hydroxyl radical (HO·), and superoxide anion (O_2−_·). For type II reaction, the triplet PS undergoes energy exchange with triplet ground state oxygen, leading to the formation of singlet oxygen ^1^O_2_. Type I and type II reactions can occur at the same time during irradiation. Nevertheless, type II reaction is mainly involved in antimicrobial photodynamic action. The reaction depends most importantly on PS used and the concentration of O_2_ in aPDT.

PS, light, and oxygen in tissue or in a cell are the three key elements of PDT, and none of them is toxic or cell/tissue damaging by itself. Upon excitation by light containing the absorption peaks of a PS (usually red or blue light, near-infrared light and even sunlight [13]), the PS transforms from ground state to the short-lived singlet state, and then relaxed to the triplet state (PS*) (Figure 3) [67]. After achieving the triplet state of a PS, two kinds of reaction follow. In the type I reaction, the excited PS reacts through electron transfer with biomolecules, such as lipids, proteins, and amino acids, to yield the superoxide anion radical (O_2_•−) and hydroxyl radical (•OH). O_2_•− undergoes dismutation to form hydrogen peroxide (H_2_O_2_), the precursor of the highly reactive •OH. •OH is extremely chemically reactive to almost all biological molecules [68]. In the type II reaction, the excited PS yields singlet oxygen (^1^O_2_) through a direct energy transfer to molecular oxygen. Like the hydroxyl radical, ^1^O_2_ is highly reactive [67]. These two types of reactions compete with each other, and the type II reaction is believed to be the principal mechanism of O_2_-dependent PDT [69]. In a microorganism, the photodynamic actions should take place where the PS deposited, as the half-life of singlet oxygen and ROS are only within microseconds and the diffusion distance is within micrometers [70]. Therefore, PDT targets multiple organelles in a cell. No evidence of any PDT-resistant microorganisms has been reported so far. On the contrary, MRSA was reported to become more sensitive to antibiotics after ICG-mediated PDT, which was partly related to mecA complex gene deletion [16].

## 6. Antimycotic Photodynamic Therapy

Most published studies of antimycotic PDT today focus on in vitro investigations [72,73]. Table 2 summarizes the clinical applications of aPDT against fungal keratitis. PSs used in aPDT for IK include toluidine blue O (TBO), methylene blue (MB) [74,75], rose bengal (RB) [20], and riboflavin (RBF) [76,77]. The following section focuses on PSs in antimycotic studies.

### 6.1. First-Generation Photosensitizers

#### Porphyrins

The modern era of PDT began in 1978 after Dougherty et al. purified hematoporphyrin derivatives and produced Photofrin, the first clinically approved PS for the treatment of human cancer [13]. The photodynamic action of hematoporphyrin was discovered on yeast cells as early as 1981 [84]. The uptake of porphyrins by *Candida* is influenced by culture conditions, and the damage caused by porphyrin-mediated PDT is determined by the metabolic activities after irradiation [84]. Most of the porphyrin PSs are highly hydrophobic. Carre et al. investigated a series of natural meso-arylglycosylporphyrins with an amphiphilic character to improve cell permeation and found that antifungal activity is correlated to PS permeation into cells [85]. A recent study showed that TMPyP [5,10,15,20-tetrakis(1-methylpyridinium-4-yl)-porphyrin tetra p-toluenesulfonate], a water-soluble porphyrin, accumulates in *C. albicans* cell wall before irradiation, which makes it unlikely to emerge resistance upon aPDT [86]. One of the beneficial effects of aPDT is that it targets multiple organelles in a cel. Thus, no mutagenic effect occurs on a yeast cell [87]. The effects of aPDT are reduced by human blood plasma and human serum albumin, with its mechanisms explained by the binding of porphyrins to albumin, and the quenching and scavenging of ROS by albumin. The clinical application of porphyrins is currently mainly replaced by the second-generation PSs with higher purity and better tissue selectivity.

### 6.2. Second-Generation Photosensitizers

In view of first-generation PSs which lack specificity to target cancer cells, the second-generation PSs are more effective and technically superior to first generation PSs. They are improved in purity, have a longer wavelength absorption, as well as higher photosensitivity and tissue selectivity. Many second-generation PSs are based on porphyrin and chlorin structures [88].

#### 6.2.1. Phenothiaziniums

The phenothiaziniums, such as toluidine blue O (TBO) and methylene blue (MB), can localize in the plasma membrane of yeast cells. Photodynamic action damages the plasma membrane, increasing the permeability of the membrane, thus leading to cell death [89]. De Souza et al. examined the effects of 0.1 mg/mL MB-mediated aPDT on four different species of Candia genus. After exposing planktonic *C. albicans, C dubliniensis, C. krusei*, and *C. tropicalis* to a low power 685 nm diode laser with 28 J/cm^2^, respectively, cell growth in all species was significantly inhibited by MB-PDT compared to the control groups [90]. However, the number and mass of the cells can both influence the effects of aPDT [91]. Giroldo et al. proved that a lower MB concentration (0.05 mg/mL) may also effectively inhibit *Candida* growth with the same light dosage [92]. In a study comparing the aPDT effects among MB with red LED, rose bangal with green LED, and ribloflavin (RBF) with UVA irradiation on inhibited growth of *C. albicans* biofilm, MB with red LED was the most effective treatment to inhibit growth on both staphylococcal and candidal biofilms [93].

TBO is an acidophilic metachromatic dye that has a high affinity for DNA and RNA contents (1). It has been widely used as a vital stain for mucosal lesions and in pathology because of its metachromatic property [94]. The study of TBO in aPDT on *C. albicans* dates back to 1994 when Wilson et al. showed that *C. albicans* was susceptible to the TBO/MB photodynamic approach [95]. A recent comprehensive systemic review was done by Wiench et al. where the authors analyzed 21 studies screened from 393 studies from 1997–2020 in the English literature. In comparison to other PSs (MB, malachite green, RB, riboflavin/blue light 460 nm, new MB N, curcumin, erythrosine and chlorin (e6)), the TBO-PDT effects on *C albicans* are about in the middle [96]. It was concluded that TBO-PDT clearly exhibits antifungal effects against oral *Candida* spp., but more investigations are needed to confirm its clinical efficacy.

#### 6.2.2. 5-Aminolevulinic Acid

Although not a PS itself, aminolevulinic acid (ALA) synthesizes the real photoactivable PPIX in a yeast cell [97]. Among different metabolites of ALA, metalloporphyrin and PPIX are predominantly accumulated in yeast cells and become photosensitive to visible light [98]. Monfrecola et al. evinced the growth inhibition of *C. albicans* after incubation with ALA at a concentration higher than 300 mg/mL for 3 h and irradiation (40 J/cm^2^) with polychromatic visible light from a slide projector equipped with a 150 W tungsten lamp. Plasma membrane damage was visualized under an electron microscope [99]. In addition to the pathogen damage, ALA-mediated PDT also inhibits virulence factors and reduces in vivo pathogenicity [100]. The treatment can also prolong survival in fungus-infected (*Fonsecaea monophora) Galleria mellonella* larvae by positively regulating its humoral immunity against infection [101].

ALA-PDT can also inhibit *C. albicans* biofilm in vitro after incubating the fungi in 15 mM ALA for 5 h followed by exposure to 300 J/cm^2^ red light [102]. However, skin damage can occur if the protocol for skin neoplastic lesions (75 J/cm^2^ irradiation after 20% ALA occluded for 4 h) is used to treat interdigital mycosis of the feet [72]. Nevertheless, with the advancing knowledge in aPDT, promising results of ALA-PDT for human fungal infections on superficial dermatophytosis and onychomycosis, deep fungal infections have been reported since the 2000s [73]. No English language reports of treating fungal keratitis with ALA-PDT have been recorded so far.

#### 6.2.3. Phthalocyanines

Phthalocyanines are the most important colorants developed in the 20th century [103]. They are analogs of two natural porphyrins, hemoglobin and chlorophyll. Phthalocyanine was discovered in 1907, and alongside its copper salts became commercially available in the 1930s as a blue color chromogen. The compound phthalocyanine is just as large as porphyrins. The parental compound has little use in PDT, yet its derivative metal complexes make viable PSs. In the study of testing sulfonated aluminum phthalocyanines (AlPcS_n_) in the context of photochemotherapy, AlPcS_2_ is found to have the highest photosensitivity compared to AlPcS_1_, AlPcS_3_, and AlPcS_4_ [104]. Among the phthalocyanine derivatives, zinc(II)-phthalocyanine (Zn(II)Pc) has the highest uptake in *C. albicans*. In comparison to the lipophilic ZnPc, the water-soluble sulphonated derivative ZnPcS binds more tightly on plasma membranes in both *Streptococcus faecium* and *C. albicans*, and causes cell membrane damage after photoactivation [105]. Phthalocyanine derivatives Zn(II)Pc, ZnPc, and ZnPc-TiO2 exhibit fungicidal effects on *C. albicans* after irradiation with a white light-emitting diode (LED) light source 90 J/cm^2^ [106]. A number of phthalocyanine-based PSs have been though different phases of clinical trials for cancer [107], yet on the other hand, clinical trials of phthalocyanine-based aPDT treatment for IK have not been addressed.

#### 6.2.4. Riboflavin

Corneal cross-linking (CXL) with RBF and UVA irradiation has become a clinical treatment for corneal ectasia [108]. These ectatic changes have typically been marked by corneal thinning and an increase in the anterior and/or posterior curvatures of the cornea, often leading to high levels of myopia and astigmatism. The most common form of ectasia is keratoconus, and other forms of ectasia can be seen after laser vision correction such as LASIK [109].

**Figure 4:**
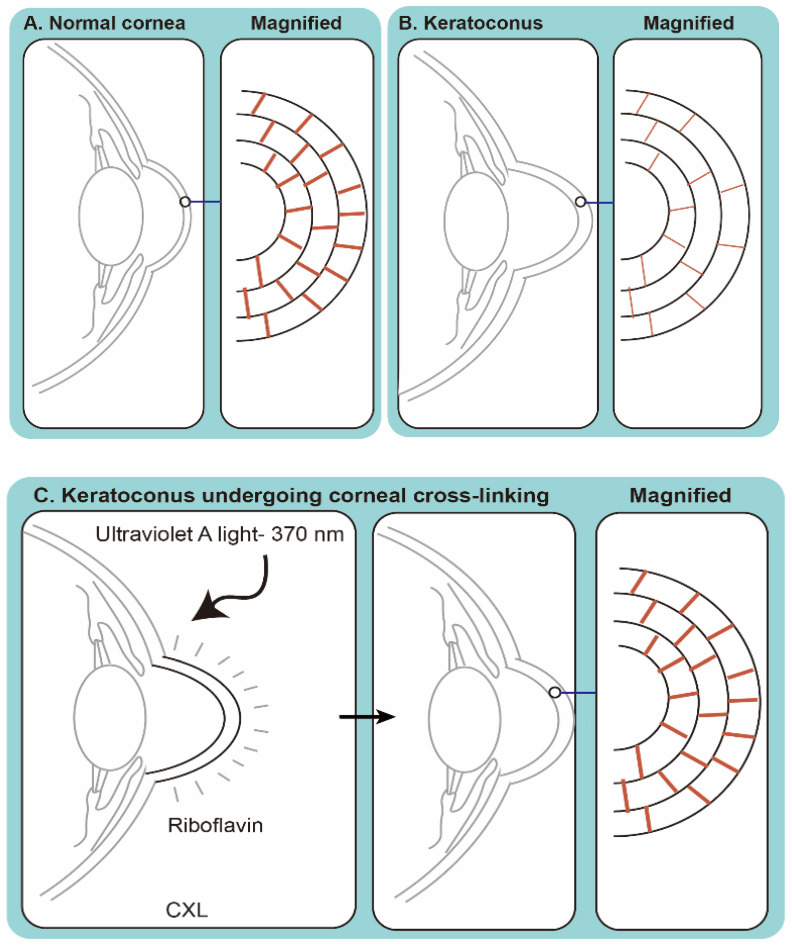
Schematic drawing of corneal crosslinking (CXL) using riboflavin as a photosensitizer and activated with UVA. (**A**) A normal corneal contour. The magnified view of the corneal stroma showed normal amount of the corneal cross-links. (**B**) Keratoconus represents a corneal disorder where central or paracentral cornea undergoes progressive thinning and steepening. Magnified view of the corneal stroma showed less cross-link bonds within the extracellular matrix of the stromal collagen (red bars). (**C**) Upon exposure of riboflavin to UV-A light, the number of covalent bonds between collagen molecules, and between collagen molecules and proteoglycans increased leading to the stiffening of the cornea. The PDT effects is mediated primarily through the generation of singlet oxygen.

As a PS, RBF generates reactive oxygen species when activated by UVA (wavelength 370 nm) to form collagen cross-links artificially (Figure 4), mainly via a type II reaction (generation of singlet oxygen) [110]. CXL was described first in the late 1990s in animal studies [111], and in 2003 Wollensak reported the first human study on CXL for keratoconus [18]. It has been approved to treat keratoconus and corneal ectasia post refractive surgery in Europe since January 2007, and in the United States since 2016 [108].

CXL is a process similar to PDT and had been used to treat IK in 2013 [112,113], with bacterial and/or fungal infections (Table 2). There are at least three potential mechanisms by which CXL may benefit patients with infectious corneal ulcers: antimicrobial, anti-inflammatory effects, and increased resistance of corneal tissue to enzymatic degradation [83]. With the increasing interest in CXL to treat IK, PACK-CXL (photoactivated chromophore for infectious keratitis), a new term was coined at the 9th CXL congress in Dublin [114].

The in vitro antimicrobial properties of PACK-CXL against common bacterial and fungal pathogens were studied in 2008 [115]. Since 2014, PACK-CXL has been applied clinically to treat severe IK, either as first-line therapy or an adjuvant therapy in a prospective clinical trial [19,107,111,112]. In a study involving 40 IK patients, the addition of PACK-CXL with continued antibiotic treatments resulted in the resolution of infections in 85% of the cases [116]. The encouraging results of another study involved 16 patients, in which PACK-CXL was used as a primary treatment for bacterial keratitis [117]. However, a recent study group found that there is no additional benefit when using PACK-CXL as an adjuvant therapy against bacterial keratitis [118].

Table 2 summarizes the clinical studies of PACK-CXL against fungal infection. Two randomized clinical trials have evaluated the efficacy of adjuvant PACK-CXL in fungal keratitis. In one trial, patients with bacterial and fungal keratitis and Acanthamoeba infestations were randomized to be treated by PACK-CXL versus medical therapy [119]. Although this trial did not identify any benefits of PACK-CXL, these results were difficult to interpret given the inclusion of different types of keratitis of a small sample size. Another small, randomized clinical trial investigated PACK-CXL as an adjuvant therapy for advanced, deep filamentous fungal ulcers and found an increased rate of perforation among those receiving PACK-CXL [78]. Later on, one retrospective series published in 2015 found no benefit to adjuvant PACK-CXL in moderate mycotic keratitis [79]. In the following year, a randomized control trial [80] evaluated the efficacy of PACK-CXL as an adjunctive therapy to treat moderate to severe IK. The results suggested that standard treatment combined with PACK-CXL does not provide additional advantageous effects, regarding the size of stromal infiltrates and corneal epithelial defect in moderate to severe IK over a 30-day period. Furthermore, a recent study evaluated the additional benefits of using PACK-CXL (0.1% RBF, 5.4 J/cm^2^) as an adjuvant therapy against fungal keratitis. This randomized controlled clinical trial consisted of four treatment arms: (1) topical natamycin 5% alone, (2) topical natamycin 5% plus PACK-CXL, (3) topical amphotericin B 0.15% alone, and (4) topical amphotericin 0.15% plus PACK-CXL. The results proved that there was no difference in infiltrate or scar size, percentage of epithelialized or adverse events in PACK-CXL plus antibiotic treatments [83].

In brief, PACK-CXL alone against fungal keratitis appears to be ineffective but might have additional benefits when combined with other treatments. Notably, a recent case report successfully eliminated the infection of post-penetrating keratoplasty multidrug-resistant *Purpureocillium lilacinum* (*Paecilomyces* sp.) keratitis by using intraoperative PACK-CXL during penetrating keratoplasty [82].

#### 6.2.5. Rose Bengal

Rose bengal (RB) is a halide derivative of fluorescein [120]. As a well-characterized dye for ophthalmic purposes, RB has long been used to enhance the visualization of corneal lesions by staining dead and devitalised cells, including mucous strands on ocular surfaces [121]. RB-mediated aPDT is able to kill *S. aureus*, *E. coli*, and *C. albicans* with roughly comparable efficiency to that of TBO [91].

In 1993, RB was demonstrated to inhibit 99% of *C. albicans* growth with photodynamic effects when combined with glutathione [122]. Another exploratory approach of using 0.1% RB as a PS with green light exposure (518 nm, 5.4 J/cm^2^) also demonstrated efficacy against multidrug-resistant *F. keratoplasticum* species [81]. Notably, in contrast to the poor visual outcome of conventional treatments against multidrug-resistant fungal keratitis [12,31], this treatment leads to a favorable result (Table 2) [81].

Furthermore, RB-mediated PDT with blue LED (455 ± 20 nm) irradiation is effective to inhibit cell growth of *C. albicans* in planktonic cultures and in biofilms [123]. Similar to PACK-CXL, it can increase resistance of corneal tissue to enzymatic degradation [124]. Interestingly, Wertheimer et al. [125] showed that corneal CXL with RB and green light is largely an oxygen dependent process compared to PACK-CXL [126]. Using enucleated deepithelialized rabbit corneas, the crosslinking procedure with 0.1% RB and green light (532 nm, 0.25 W/cm^2^, 200 J/cm^2^) produced comparable effects to PACK-CXL [127].

In a study comparing RB-mediated aPDT using green LED (518 nm) to PACK-CXL using an ultraviolet-A LED array (peak wavelength: 375 nm) to treat clinical fungal isolates (*F. solani*, *A. fumigatus*, *C. albicans*) in vitro, only RB-PDT successfully inhibited the growth of all types of fungi. The authors concluded that RB may be a promising PS compared to RBF in aPDT for fungal infection [77]. Recently, clinical applications of RB-PDT for fungal keratitis (Table 2) showed encouraging results. After RB-mediated aPDT, the anterior stromal changes with a demarcation line in a human cornea was detected with a slit lamp and anterior segment optical coherence tomography (AS-OCT). Histology showed anterior stromal scarring with disorganization of the collagen bundles at a depth of 220 μm, which suggested an efficient penetration of RB [128]. RB-mediated PDT is well tolerated in most patients.

Since its first successful case in 2017 introduced by Amescua et al. [81], RB has become a potentially effective PS to treat fungal keratitis. A case series evaluating 18 patients in 2019 confirmed the treatment effect of RB-mediated aPDT against progressive corneal infection (*Acanthamoeba*, *Fusarium* spp., *Pseudomonas*, *Curvularia* spp.) [20].

An ideal PS for a successful aPDT on fungal keratitis has a high yield of singlet oxygen after irradiation. Peterson et al. developed a singlet oxygen dosimeter detection system that can detect singlet oxygen during experimental RB-PDT using an ex vivo human eye [129]. The dosimeter will clearly help optimize future RB-PDT treatment parameters.

## 7. aPDT against Fungal Biofilm

It has been demonstrated that many fungal biofilms are susceptible to aPDT, particularly *Candida* in dental diseases [130]. An earlier study investigated the effects of Photofrin-mediated PDT (Hg arc lamp, 400–700 nm, 15 mW/cm^2^, 18 J/cm^2^) against *C. albicans* biofilms and germ tubes [131]. After exposing biofilm to PDT at 18 J/ cm^2^, a significant reduction of metabolic activity was demonstrated compared to the biofilm treated with amphotericin B (10 µg/mL) alone. The same group also obtained similar results against biofilms of *C. albicans* and *C. dubliniensis* using erythrosine (400 mM) with green LED light (532 ± 10 nm, 90 mW/cm^2^, 16.2 J/cm^2^; 237 mW/cm^2^, 42.63 J/cm^2^) with significant reductions in CFU/mL of 0.74 log and 0.21 log, respectively [131]. Similar to other PS-based aPDT, RB is less effective to treat biofilm if the light dose is low. RB (12.5 μM) with green light-emitting diode (LED) (532 nm) irradiation (16.2 J/cm^2^) fails to inhibit heterotypic biofilm formation of *C. albicans* and *B. atrophaeus* [132]. Nevertheless, the non-toxic and minimally invasive nature of aPDT supports it to be a potential strategy to control microbial biofilms in the future [133].

## 8. Challenges in Trans-Corneal Drug Delivery

**Figure 5:**
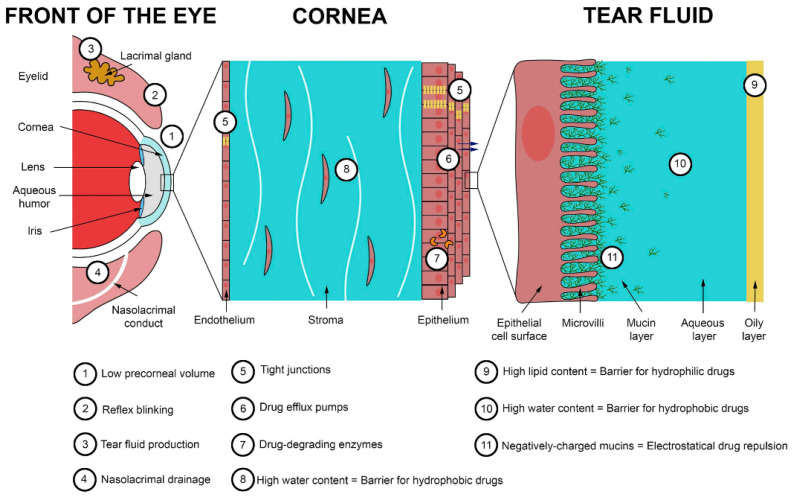
Main static and dynamic barriers for trans-corneal drug delivery [136]. Reprinted from *Journal of Controlled Release*, Volume 321, doi.org/10.1016/j.jconrel.2020.01.057. Clotilde Jumelle, Shima Gholizadeh, Nasim Annabi, Reza Dana. Advances and limitations of drug delivery systems formulated as eye drops. Reprinted with permission from ref. [136]. Copyright 2021 Elsevier.

The corneal epithelium together with the tear film provides an effective outermost barrier to prevent pathogens and environmental toxic substances like drugs from entering the eye [134,135]. The barriers for trans-corneal drug delivery are: (1) the limited volume of the eye drop that can be applied to the eye due to the limited precorneal surface area, and most of the volume applied being eliminated during blink reflex triggered by the eye drop; (2) the drugs that remained on the precorneal surface are further degraded by enzymes in the tear film, the outermost layer of the cornea (numbers 9–11 in Figure 5). The thin fluid tear film is composed of three layers: an outer oily layer, an intermediate aqueous layer, and an inner mucin layer (Figure 5). Hydrophilic and hydrophobic drugs are hindered by the two outermost layers, respectively. The drug molecules can be attracted or repulsed by the negatively charged mucins. (3) The fast turnover rate of the tear film (0.5–2.2 µL/min) is accompanied by an estimation that all active ingredients are eliminated on the corneal surface 15–25 min after application [136].

Even though the drug penetrates the tear film, it is estimated that less than 5% of drug can reach to the anterior chamber because the cornea, scleral, and conjunctival tissue are also effective barriers. The cornea includes three major layers: the epithelium, the stroma, and the endothelium. The hydrophobic corneal epithelium is composed of non-keratinized stratified squamous cells with intercellular tight junctions, which form a strong permeation barrier for hydrophilic drugs. The epithelium also contains drug efflux pumps and drug-degrading enzymes that prevent drugs from entering. The stroma contains 80% water and this limits the penetration of hydrophobic drugs. The endothelium also contains tight junctions between cells which hinder hydrophilic drugs, but to a lesser extent in comparison to the epithelium due to its lesser cell thickness.

At present, PDT is limited to treating superficial infections due to the limitation of visible light penetration and selectivity of PSs to infected tissues. Due to the poor penetration of PSs, the traditional Dresden protocol for IK treatment is comprised of the removal of the central epithelium before the repetition of RBF eye drop instillation every 3–5 min for 30 min, and a continued application during the UV irradiation at 365 nm and 3 mW/cm^2^ for 30 min [18]. The reason behind repetitive application is to achieve an adequate concentration of RBF or RB in corneal stroma [137]. Nevertheless, all of the current protocols are time-consuming. Postoperative pain and poor corneal healing are also common disadvantages of this procedure. The protocol has been modified into transepithelial (epi-on) and accelerated CXL with a higher-powered light source and a shorter treatment time. Wollensak et al. found that drug penetration in epithelium-on protocol was less successful in comparison to epithelium-off protocols, and its effectiveness requires further studies to confirm [124]. Moreover, a randomized controlled trial found that around one-fifth of epithelium-on PACL-CXL cases resulted in the progression of keratoconus versus none in the epithelial-off cases at one-year follow-up [125].

The consensus in the ophthalmology community is to use an overall fluence of 5.4 J/cm^2^ in PACK-CXL, however the standard Dresden protocol remains the mainstream. A slow irradiation may allow improved oxygenation during PDT. Another concern is the depth of drug penetration. Using enucleated and de-epithelialized rabbit eyes, the depth of penetration of RBF or RB was less than 200 μm [124]. Deeper drug penetration can be achieved using the ultrasound energy [138] and iontophoresis [139]. Wu et al. found iontophoresis to be a safe and effective method to improve PACK-CXL in an epi-on protocol to treat keratoconus when 21 eyes of 12 patients improved in terms of keratoconus, visual acuity, corneal tomography, and morphological alteration in the corneal stroma at 24 months after the procedure [140].

## 9. The Future of aPDT in the Era of Nanomedicine

The future research direction of aPDT includes exploring PSs other than RBF and RB for fungal keratitis. Different strategies have been applied to improve ocular drug delivery, including the use of penetrating-enhancing compounds (cyclodextrins, chelating agents, crown ethers, bile acids and bile salts, cell-penetrating peptides, and other amphiphilic compounds) [135], microemulsions [141], and the incorporation into nanoparticles [142,143]. The incorporation of nanotechnology to facilitate the delivery, efficiency, and visualization of PSs in aPDT [130,144,145] may advance the treatment profoundly. With the aid of nanotechnology, a PS can be modified for slow release and at the surface for target delivery, to increase in situ oxygen, elongate absorption peak to treat deeper tissue, and to simultaneously diagnose disease and provide treatment (theranostics). For example, Zhang’s team [144] used upconversion nanoparticles encapsulating two PSs (MC540 and ZnPc) in PDT to treat melanoma cells effectively in vitro and in vivo. After irradiation with a near-infrared (NIR) 980-nm laser matrix, the nanoparticles were able to efficiently upconvert the energy to green (∼540 nm) and red (∼660 nm) visible light wavelengths and transfer it to the encapsulated PSs. Recently, Tezuka et al. designed a biodegradable nanoparticle that encapsulates a hydrophobized rose bengal (RB) derivative for NIR-induced upconversion PDT [145]. The nanoparticles exhibited high singlet oxygen yield and high selectivity to cancer cells. In addition, hind limb blood vessels and the liver could be visualized under a NIR camera by the fluorescence (wavelength: 1550 nm) after intravenous injection, suggesting a simultaneous imaging and therapy for a “see and treat” approach. This technology may be applied to resolve the problem of poor penetration of antifungal agents for deep fungal stromal keratitis.

## 10. Conclusions

The results of in vitro investigations have demonstrated the potential of aPDT on fungal keratitis. Importantly, aPDT destroys fungal cells non-selectively. Thus, there have been no reports of PDT-resistance and/or cases of fungi becoming drug-resistant after aPDT treatments. Moreover, aPDT associated genotoxic or mutagenic effects to fungal or human cells have so far not been observed. Nonetheless, despite the success of many in vitro studies, animal studies and human trials are indispensable. At the time of the current review, RB appears to be a viable potential PS to treat fungal keratitis. A novel approach of PS delivery via the assistance of nanotechnology may be the next promising development for multidrug-resistant fungal keratitis.

## Figures and Tables

**Table 1:** Outcomes of case reports affected by multidrug resistant fungal keratitis.

Ref. (Year) Citation	Pathogens	Initial VA	Antifungal Drugs	Surgery	Outcome
Sponsel (2002) [30]	*F. solani*	Not mentioned	AMB-intravenous, topical KTC-topical NAT-topical POS-PO, topical	PK	VA: 6/30
Guarro (2003) [53]	*F. polyphialidicum*	1/200	AMB-topical	Corneal transplantation	VA: 20/40 (improved)
Tu (2007) [54]	*F. solani*	HM	AMB-IVI, topical FLC-PO ITC-PO NAT-topical POS-PO VRC-intravenous, IVI, PO	PK for 3 times	VA: CF (improved)
	*Fusarium* sp.	Not mentioned	AMB-topical FLC-PO NAT-topical VRC-PO, topical POS-PO, topical	PK for 2 times	Resolution of inflammation
	*Fusarium* sp.	Not mentioned	AMB-AC injection, topical CYA-topical FLC-PO NAT-topical POS-PO VRC-IVI, PO, topical	PK, penetrating patch graft	Poor vision, awaiting repeat corneal transplantation
Proença-Pina (2010) [55]	*F. solani*	HM	AMB-AC irrigation, topical VRC-PO, topical	PK	VA: 20/50 (improved)
Edelstein (2012) [56]	*F. solani*	HM	AMB-ICI, IVI, topical FLC-PO ITC-PO NAT-topical POS-PO VRC-PO, topical	PK for 2 times, pars plana vitrectomies, enucleation	Enucleation
Antequera (2015) [31]	*F. solani*	-	AMB-intravenous CAS-intravenous VRC-intravenous, PO, topical	Enucleation	Enucleation
Sara (2016) [12]	*F. solani*	6/12	AMB-IVI NAT-topical VRC-IVI, PO, topical	PK, enucleation	Enucleation

AC: Anterior chamber; AMB: Amphotericin B; CAS: Caspofungin; CF: Counting fingers; CYA: Cyclosporine A; FLC: Fluconazole; HM: Hand movement; ICI: Intracameral injection; ITC: Itraconazole; IVI: Intravitreal injection; KTC: Ketoconazole; NAT: Natamycin; PK: Penetrating keratoplasty; PO: oral; POS: Posaconazole; VA: Visual acuity; VRC: Voriconazole.

**Table 2:** Clinical reports of antimycotic photodynamic therapy for fungal keratitis.

Ref. (Year) Citation	Pathogens	Study Type	Case Number	Photosensitizer	Light Source (Wavelength), Irradiance, Irradiation Time or Radiant Exposure	Outcome
Iseli (2008) [19]	*Acremonium* sp.	Case reports	1	0.1% RFB	UVA 3.0 mW/cm^2^ 30 min	VA: CF after CXL, 20/30 after DALK (8 months after CXL) (improved)
	*Fusarium* sp.		1	0.1% RFB	UVA 3.0 mW/cm^2^ 30 min	Corneal infiltrate progressed after CXL → PK
Uddaraju (2015) [78]	*Aspergillus* sp., *Fusarium* sp.	RCT	6	0.1% RFB	UVA (370 nm) 3.0 mW/cm^2^ 30 min	VA: HM (2 out of 6 cases), LP (2 out of 6 cases), 6/60 (2 out of 6 cases) (~20% cases improved; ~20% cases stable disease, ~60% cases worsened)
Vajpayee (2015) [79]	*Aspergillus* sp., *Fusarium* sp.	Retrospective study.	20	0.1% RFB	UVA (365 nm) 3.0 mW/cm^2^ 30 min	BCVA: 1.13 ± 0.55 (stable disease)
Kasetsuwan (2016) [80]	*Fusarium* sp., *Aspergillus* sp., *Purpureocillium* sp., *Pythium* sp.	RCT	8	0.1% RFB	UVA (365 nm) 3.0 mW/cm^2^ 30 min	Median size of stromal infiltration: 30.2 mm^2^→ 9.1 mm^2^ Median size of epithelial defect: 23.7 mm^2^→ 1.42 mm^2^
Amescua (2017) [81]	*Fusarium* sp.	Case reports	1	0.1% RB	Green light LED (518 nm) 0.9 J/cm^2^→ 1.8 J/cm^2^	Clear cornea with fine endothelial function
Mikropoulos (2019) [82]	*P. lilacinum*	Case report	1	RFB	UVA 9.0 mW/cm^2^ 30 min (intraoperative)	VA: CF at 1 m (stable disease)
Naranjo (2019) [20]	*Fusarium* sp.	Consecutive case series.	4	0.1% RB	Green light LED 6.0 mW/cm^2^ 15 min	BCVA: 20/100, 20/800, HM, NLP (50% cases improved; 25% cases stable disease, 25% cases worsened)
	*Curvularia* sp.		1	0.2% RB	Green light LED 6.0 mW/cm^2^ 15 min	BCVA: 20/50 (improved)
Prajna (2020) [83]	*Aspergillus* sp., *Bipolaris* sp., *Colletotrichum* sp., *Curvularias* sp., *Exserohilum* sp., *Fusarium* sp., *Scedosporium* sp.	RCT	55	0.1% RB	UVA (365 nm) 3.0 mW/cm^2^ 30 min	VA: 3.2 Snellen lines worse at 3 months than baseline VA (worsened in all cases)

BCVA: Best-corrected visual acuity; CF: Counting fingers; CXL: Corneal crosslinking; DALK: Deep anterior lamellar keratoplasty; HM: Hand movement; LED: Light emitting diodes; LP: Light perception; NLP: No light perception; PK: Penetrating keratoplasty; RB: rose bengal; RCT: Randomized controlled trial; RFB: riboflavin; UVA: Ultraviolet A; VA: Visual acuity.

## Data Availability

All data was presented in the manuscript.
